# MSCs derived from amniotic fluid and umbilical cord require different administration schemes and exert different curative effects on different tissues in rats with CLP-induced sepsis

**DOI:** 10.1186/s13287-021-02218-8

**Published:** 2021-03-06

**Authors:** Rui Chen, Yingjun Xie, Xuan Zhong, Fei Chen, Yu Gong, Na Wang, Ding Wang

**Affiliations:** 1 Department of Obstetrics and Gynecology, Key Laboratory for Major Obstetric Diseases of Guangdong Province, The Third Affiliated Hospital of Guangzhou Medical University, Guangzhou 510150 Guangdong, China; 2 Key Laboratory of Reproduction and Genetics of Guangdong Higher Education Institutes, The Third Affiliated Hospital of Guangzhou Medical University, Guangzhou 510150 Guangdong, China; 3 Medical Intensive Care Unit, Guangdong Women and Children Hospital, Guangzhou 510150 Guangdong, China; 4 Central Laboratory, The Third Affiliated Hospital of Guangzhou Medical University, Guangzhou 510150 Guangdong, China; 5 Department of Pathology, The Third Affiliated Hospital of Guangzhou Medical University, Guangzhou 510150 Guangdong, China

**Keywords:** Mesenchymal stem cells, Sepsis, Amniotic fluid, Umbilical cord, Curative effect

## Abstract

**Background:**

Mesenchymal stem cells (MSCs) are derived from multiple tissues, including amniotic fluid (AF-MSCs) and the umbilical cord (UC-MSCs). Although the therapeutic effect of MSCs on sepsis is already known, researchers have not determined whether the cells from different sources require different therapeutic schedules or exert different curative effects. We assessed the biofunction of the administration of AF-MSCs and UC-MSCs in rats with caecal ligation and puncture (CLP)-induced sepsis.

**Methods:**

CLP was used to establish a disease model of sepsis in rats, and intravenous tail vein administration of AF-MSCs and UC-MSCs was performed to treat sepsis at 6 h after CLP. Two phases of animal experiments were implemented using MSCs harvested in saline with or without filtration. The curative effect was measured by determining the survival rate. Further effects were assessed by measuring proinflammatory cytokine levels, the plasma coagulation index, tissue histology and the pathology of the lung, liver and kidney.

**Results:**

We generated rats with medium-grade sepsis with a 30–40% survival rate to study the curative effects of AF-MSCs and UC-MSCs. MSCs reversed CLP-induced changes in proinflammatory cytokine levels and coagulation activation. MSCs ameliorated CLP-induced histological and pathological changes in the lung, liver and kidney. AF-MSCs and UC-MSCs functioned differently in different tissues; UC-MSCs performed well in reducing the upregulation of inflammatory cytokine levels in the lungs and inhibiting the inflammatory cell infiltration into the liver capsule, while AF-MSCs performed well in inhibiting cell death in the kidneys and reducing the plasma blood urea nitrogen (BUN) level, an indicator of renal function.

**Conclusions:**

Our studies suggest the safety and efficacy of AF-MSCs and UC-MSCs in the treatment of CLP-induced sepsis in rats and show that the cells potentially exert different curative effects on the main sepsis-affected tissues.

## Background

Sepsis is defined as a systemic inflammatory response syndrome (SIRS) that is induced by an infection, leading to an imbalance in the adaptive and innate immune systems in an individual. Sepsis is a heterogeneous syndrome caused by different pathogens and different effect sites that collectively result in sustained excessive inflammation and immune suppression within individuals [1]. In the hyperinflammatory phase of sepsis, the innate and adaptive immune systems are activated to eliminate the pathogens causing the disease [2]. Medical efforts such as the Surviving Sepsis guidelines [3] have been implemented to improve critical care, early detection and rapid intervention, resulting in a reduction in the mortality of sepsis in recent years, but the mortality rate is still approximately 20% [4, 5]. Some therapeutic strategies that target key events in the immunopathology of sepsis have been successful in reducing mortality and improving the clinical syndrome of sepsis in models and ongoing clinical trials, including peptide [6], cytokine [7, 8], antibody [9] and cell-based [10] therapies.

Mesenchymal stem cells (MSCs) are adult stem cells, so named because they were first derived from bone marrow [11]. Over the past decade, MSCs have become a topic of increasing interest in the scientific community because these cells are regarded as a potential tool in regenerative medicine and cell therapy. They can be extracted from both healthy donors and patients and are easily expanded in vitro to a therapeutic volume without substantial ethical concern. MSCs not only have been used as regeneration agents [12] but also can modulate immune responses in different inflammatory microenvironments [13] and relieve cell death and tissue injury in pathological and physiological states [14]. Clinical evidence for the treatment of COVID-19 with MSCs suggests that it might be considered for compassionate use in critically ill patients to reduce morbidity and mortality [15]. According to the literature, MSCs indeed reduce mortality in different animal models of sepsis [10], and several clinical trials administering allogeneic MSCs to treat sepsis and septic shock have been registered at *clinicaltrials.gov*. The safety and efficacy of MSCs should be explicitly determined before their large-scale clinical application, and the study of MSC biofunction in sepsis pathology would benefit from acquiring an advanced application scheme for the clinical setting.

In this study, we provide evidence that intravenous tail vein injections of human amniotic fluid MSCs (AF-MSCs) and UC-MSCs are both therapeutic programmes that reduce mortality in rat models of caecal ligation and puncture (CLP)-induced sepsis. Notably, we documented that the curative effects of these MSCs included a reduction in the infection-induced increase in inflammatory cytokine levels, reductions in pathological tissue damage and cell apoptosis and the prevention of the functional deterioration of the tissues. The cellular therapeutic effects of AF-MSCs and UC-MSCs on rats with sepsis reported here provide a new perspective for the study of the biological effects of these MSCs on different tissues in a sepsis model.

## Methods

### Study design

The study was approved by the Academic Committee of the Third Affiliated Hospital of Guangzhou Medical University. Animal experiments were approved by the Institutional Animal Care and Use Committee of Guangzhou Medical University. The rat CLP model was used as the disease model of sepsis, and intravenous tail vein administration of AF-MSCs and UC-MSCs was performed to treat sepsis at 6 h after CLP. We established two phases of animal experiments to determine the effects of MSCs with and without filtering through a 70-μm filter on a rat model of CLP-induced sepsis. The amounts of AF-MSCs and UC-MSCs administered were 1 × 10^6^ cells in 150 μl.

#### Phase 1

MSCs were harvested in saline without filtration. The following four groups were established in this phase: normal saline sham (*n* = 3), sepsis control (*n* = 7), sepsis + AF-MSCs (*n* = 7) and sepsis + UC-MSCs (*n* = 7) groups. A statistical analysis of the mortality of the rats in each group over a 72-h period was performed.

#### Phase 2

MSCs were harvested in saline and filtered. The groups were established as in Phase 1. In this phase, the number of rats in each group was increased, and physiological and biochemical analyses, as well as statistical calculations of mortality, were conducted (9 rats in the sham group, 21 rats in the sepsis group, 21 rats in the sepsis + AF-MSC group and 16 rats in the sepsis + UC-MSC group). Six surviving rats from each group were sacrificed at 24 h after CLP for physiological and biochemical analyses, and the remaining rats in each group were used for the survival analysis at 72 h. The levels of oedema in the lungs were analysed in 3 rats, leaving 3 rats for further lung analyses. The liver and kidney tissues of 5 rats were analysed.

### Cell culture

The derivation, maintenance and expansion of MSCs were performed using previously published protocols [16]. Briefly, UC-MSCs and AF-MSCs were maintained in low-glucose Dulbecco’s modified Eagle’s medium (CORNING, Cat# 10014CVR, Virginia, USA) supplemented with 15% foetal bovine serum (FBS) (Gibco, Thermo Fisher Scientific, Cat# 10099141, CA, USA), 100 U/ml penicillin and 100 μg/ml streptomycin (Gibco, Cat# 15140122) at 37 °C and 5% CO_2_. The cells were harvested and passaged with trypsin-EDTA (Gibco, Cat# 25300054) upon reaching 70–80% confluence.

### Caecal ligation and puncture

Adult (8–10 weeks) male Sprague-Dawley rats (Animal Experimental Center of Southern Medical University, Guangzhou, China) were randomly assigned to the 4 study groups. Rats with medium-grade sepsis were produced by CLP using a protocol [17], with some modifications. Briefly, the rats were anaesthetized with an intraperitoneal injection of 50 mg/kg pentobarbital, to ensure that the rats did not wake up before the surgical operation was complete. The abdomen of the anaesthetized rats was shaved, and the skin disinfected prior to performing a 2-cm-long midline laparotomy incision. The caecum was identified, gently exteriorized, ligated at approximately one third of its length using silk sutures, and then punctured with a 21-gauge needle in three locations of the ligated section. Finally, the caecum was replaced in the abdominal cavity, and the abdominal musculature and skin were stitched up. The rats in the sham group received laparotomy and caecum exteriorization without ligation or puncture. Antibiotics and fluid resuscitation were not administered during the study.

### Lung wet and dry weights

Six rats from each group were sacrificed at 24 h, and three were randomly selected for an analysis of the wet and dry weights of the lung. The wet weight was determined by weighing the lung immediately following dissection, while the dry weight was determined by weighing the lung after drying in a 65 °C incubator for 2 days.

### Histology and tissue staining

Histological study and staining of organs from each study group were conducted, including the right lung, liver and kidney. The organs were fixed with 4% paraformaldehyde, dehydrated and embedded in paraffin. Specimens were cut into paraffin sections for further assays. Haematoxylin–eosin (HE) staining (Cat#G1121, Solarbio, Beijing, China) was performed to identify general histology. Masson staining (Cat# G1345, Solarbio) was carried out to label collagen in the connective tissue. Periodic acid–Schiff (PAS) staining (Cat# G1281, Solarbio) was utilized to identify glycogen accumulation within liver tissue. TdT-mediated dUTP Nick-End Labelling (TUNEL) (Cat# G3250, Promega (Beijing) Biotech Co., Beijing, China) was used to label dead cells, according to the manufacturer’s instructions. All pictures were recorded by a microscope (BK6000, CNOPTEC, Chongqing, China).

### Biochemical assay

The concentration of immune cytokines in the serum and bronchoalveolar lavage fluid (BALF) were determined by enzyme-linked immunosorbent assay (ELISA) kits purchased from CUSABIO BIOTECH (Wuhan, Hubei, China) according to the manufacturer’s instructions, including IL-1β (Cat# CSB-E08055r), IL-6 (Cat# CSB-E04640r), IL-10 (Cat# CSB-E04595r) and TNF-α (Cat# CSB-E11987r). Coagulation-related biochemistry was assessed using commercial kits from the Instrumentation Laboratory (Bedford, MA, USA) on an ACLTOP700 analyser (Werfen, Beijing, China) according to the manufacturer’s instructions; the reported parameters included prothrombin time (PT) (RecombiPlasTin 2G, Cat# 0020003050), activated partial thromboplastin time (APTT) (SynthASil kit, Cat# 0020006800) and plasma fibrinogen (FIB) (Fibrinogen-C XL, Cat# 0020003900). Tissue function-associated biochemistry was assessed using commercial kits from Roche (Mannheim, Germany) on a COBAS C702 instrument according to the manufacturer’s instructions and included aspartate aminotransferase (AST) (Cat# 05850819190), alanine aminotransferase (ALT) (Cat# 05850797190), blood urea nitrogen (BUN) (Cat# 05171873190) and creatinine (Cat# 05168589190).

### Statistical analysis

Quantitative results of experiments were expressed in a scatter plot, unpaired Mann-Whitney test were applied to compare the difference between two groups and Tukey’s test was used for comparisons among multiple groups. The statistical log-rank test (Mantel-Cox) was used to compare the survival data of the different groups. *P* values < 0.05 were considered statistically significant.

## Results

### AF-MSCs and UC-MSCs rescue CLP-induced sepsis in adult rats

**Fig. 1:**
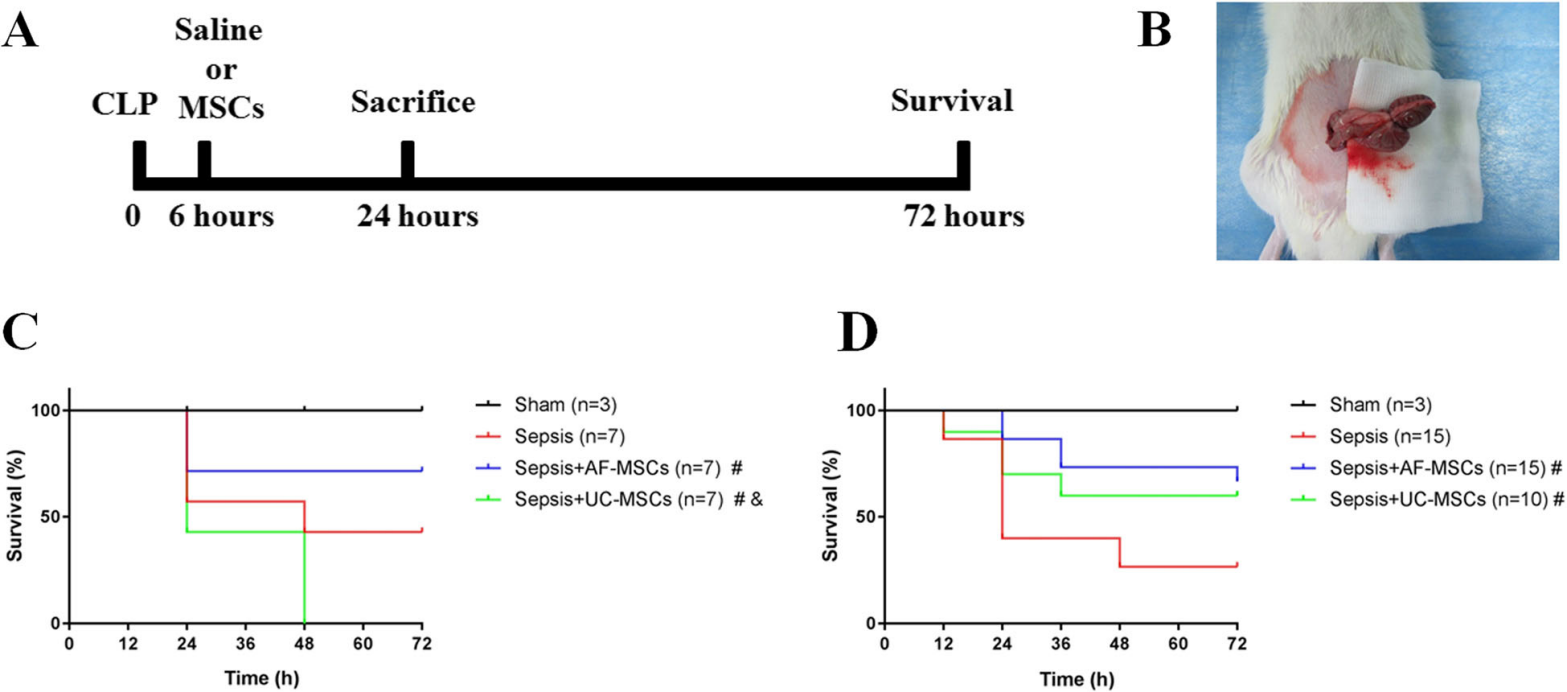
The administration of MSCs improved mortality in adult rats with CLP-induced sepsis, and UC-MSCs must be filtered to exert beneficial effects. Schematic representation of the time course of the rodent model of CLP-induced sepsis, MSC treatments and data collection (**a**). Generation of the rat model with medium-grade sepsis using CLP (**b**). The survival curves of rats that underwent CLP and received different MSC therapies without 70-μm filtration (**c**). *n* = 3 rats in the sham group and *n* = 7 rats in the other groups; ^#^*p* < 0.05 compared with the sepsis group; ^&^*p* < 0.05, compared with sepsis+AF-MSC group. The survival curves of rats that underwent CLP and received different MSC therapies with 70-μm filtration (**d**). *n* = 3 rats in the sham group and *n* = 10–15 rats in the other groups; ^#^*p* < 0.05 compared with the sepsis group

Adult Sprague-Dawley rats that underwent CLP were established as a sepsis model and treated with different MSCs resuspended in saline or saline alone 6 h after CLP surgery. Six surviving individuals from each group were sacrificed for histological and biochemical analyses at 24 h, and the survival rate of the remaining rats was determined at 72 h and a statistical analysis was performed (Fig. 1a). We generated rats with medium-grade sepsis with 60–70% mortality (Fig. 1b). Two phases of animal experiments were implemented with the harvested MSCs in saline with or without filtration through a 70-μm diameter filter. The administration of AF-MSCs without filtration significantly increased the survival rate, while sepsis rats treated with UC-MSCs exhibited 100% mortality (Fig. 1c). The administration of filtered MSCs significantly increased the survival rate, and a significant difference was not observed between the AF-MSC and UC-MSC treatments (Fig. 1d).

### MSCs reverse CLP-induced changes in proinflammatory cytokine levels and coagulation activation

**Fig. 2:**
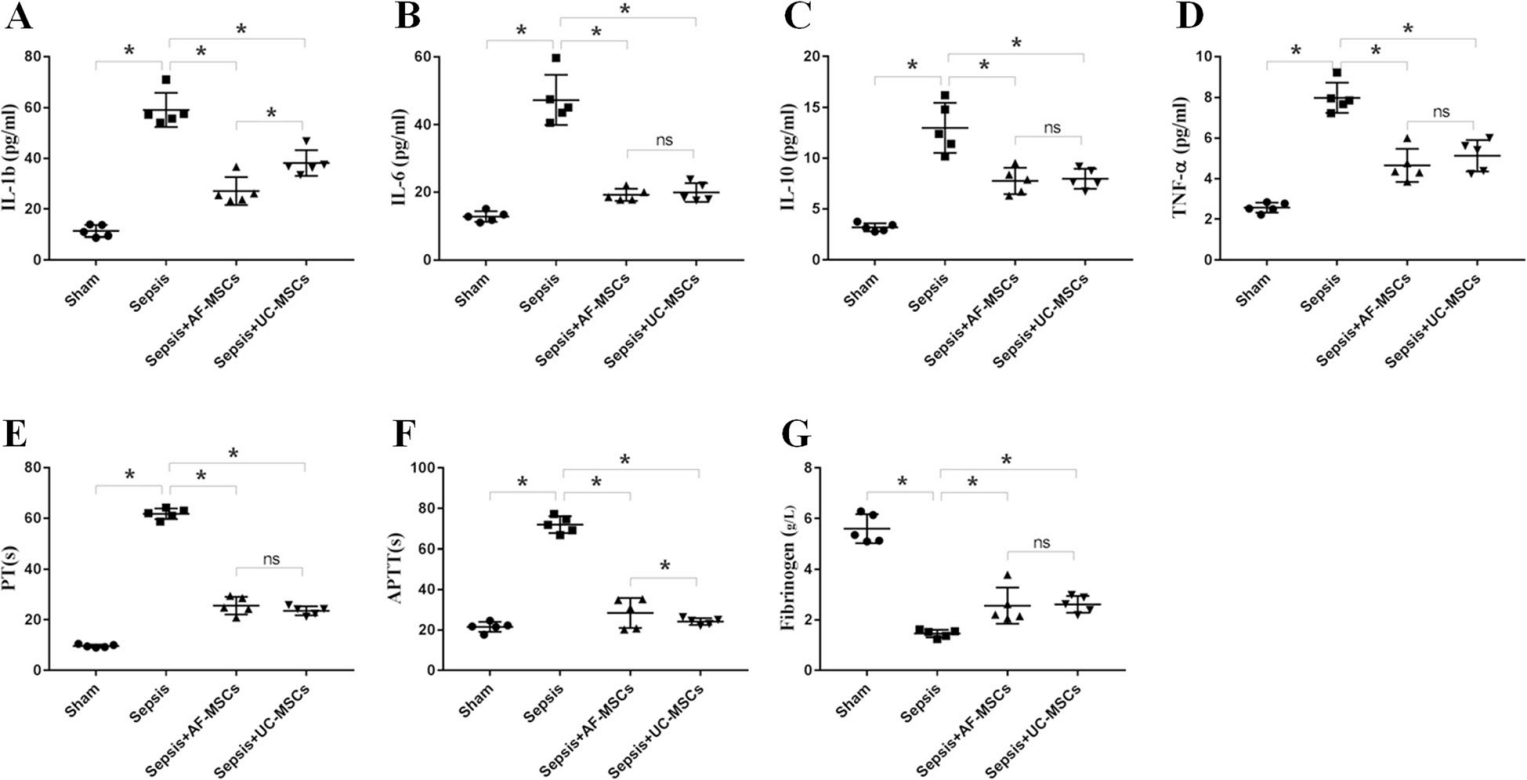
The administration of MSCs reversed CLP-induced blood plasma changes in the septic rats. The plasma levels of the following proinflammatory cytokines were measured: IL-1β (**a**), IL-10 (**b**), IL-6 (**c**) and TNF-α (**d**). Compared with the sham treatment, CLP induced the upregulation of these cytokines, while compared with the sepsis treatment, MSC administration downregulated these cytokines. Coagulation activation was assessed by determining PT (**e**), APTT (**f**) and fibrinogen levels (**g**). Compared with the sham treatment, CLP increased the PT and APTT and decreased fibrinogen levels, and these changes were reversed by the administration of MSCs. PT, prothrombin time; APTT, activated partial thromboplastin time. *p < 0.05; ns, no significant difference

Six rats from each group in the second round of the animal experiment were sacrificed for further biological analyses. For blood pathology, we assessed changes in the serum levels of proinflammatory cytokines and coagulation activation parameters. The serum concentrations of four typical inflammatory cytokines were measured, namely, IL-1β, IL-10, IL-6 and TNF-α (Fig. 2a–d). Compared to the sham treatment, CLP induced significant increases in the levels of these cytokines, and compared with the sepsis control treatment, both of the MSC treatments led to a significant decrease. The activation of coagulation induced by sepsis and the MSC treatments was measured by determining the prothrombin time (PT), activated partial thromboplastin time (APTT) and plasma fibrinogen level (Fig. 2e–g). A significant increase in the PT and APTT was observed in the sepsis group compared with the sham group, and a significant decrease was detected in the MSC-treated groups compared with the sepsis group. Additionally, MSC treatments significantly reversed the CLP-induced decrease in plasma fibrinogen levels. No definite trend of reversal in the CLP-induced pathological changes in the blood was observed in the comparison of the AF-MSC and UC-MSC groups.

### MSCs ameliorate CLP-induced histological changes, oedema, cell death and proinflammatory cytokine levels in the lung

**Fig. 3:**
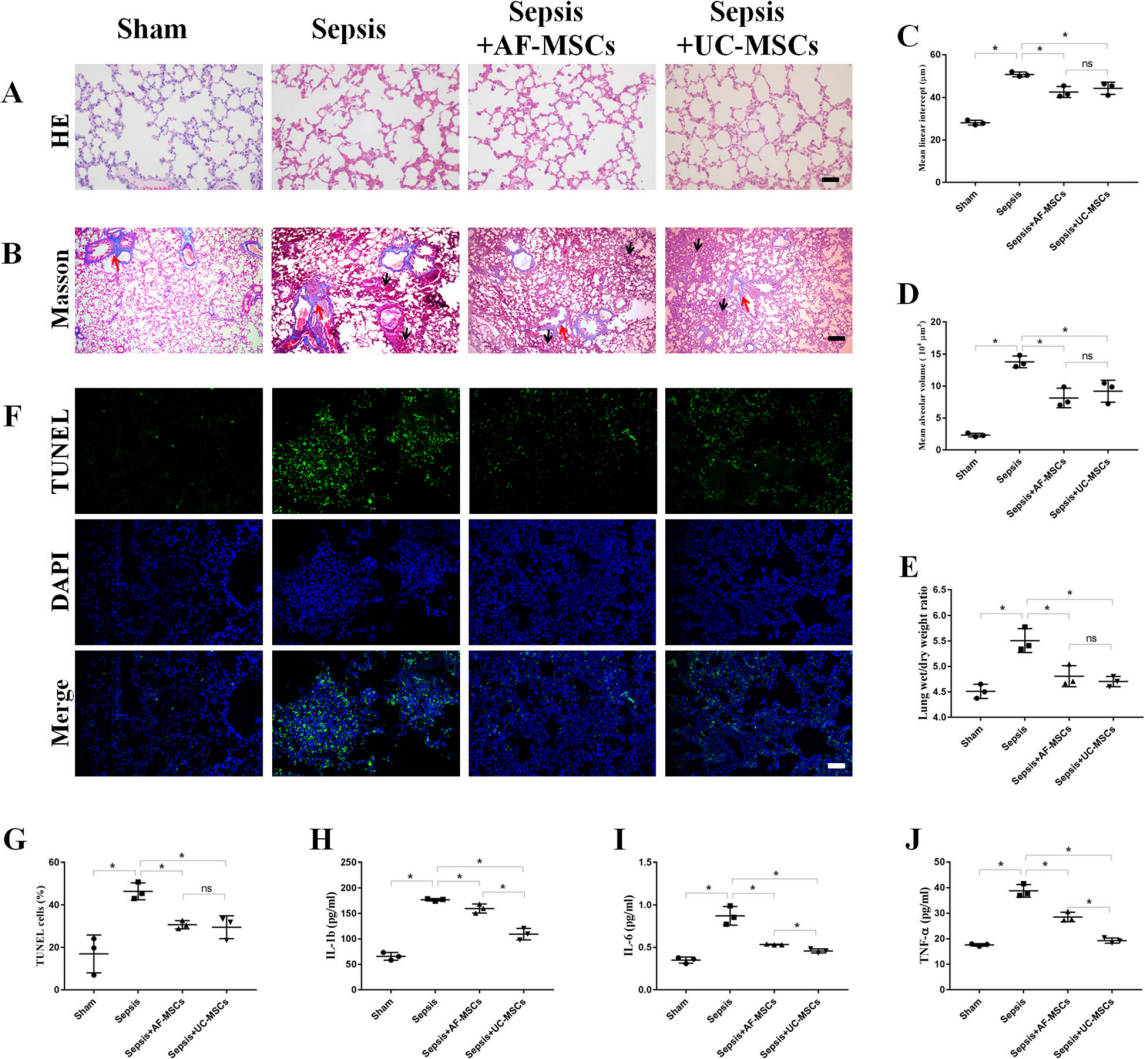
The administration of MSCs ameliorated CLP-induced pathological changes in the lungs of septic rats, while UC-MSCs were more effective at reducing proinflammatory cytokine levels than AF-MSCs. The histological changes in the lung were identified using HE (**a**) and Masson’s trichrome (**b**) staining. Both types of MSCs ameliorated CLP-induced alveolar expansion, according to the mean linear intercept (**c**) and mean alveolar volume (**d**). Other histological changes included irregular changes in the bronchial tube (red arrow) and interstitial hyperplasia (black arrow) in the sepsis group, and these changes were partially ameliorated in the MSC groups. Lung oedema, cell death and upregulation of the inflammatory cytokines due to sepsis were ameliorated by treatment with MSCs from both sources, according to wet to dry weight ratio (**e**), TUNEL staining (**f** and **g**) and IL-1β (**h**), IL-10 (**i**) and TNF-α (**j**) levels. For **a** and **f**, scale bar = 20 μm; for **b**, scale bar = 100 μm. *p < 0.05; ns, no significant difference

CLP-induced sepsis involving the lung causes histological changes, induces oedema, increases cell death and upregulates the levels of proinflammatory cytokines. The administration of both types of MSCs attenuated these changes, and UC-MSCs were much more successful in reducing inflammatory cytokine expression than AF-MSCs. Sepsis-induced histological changes and the ability of MSCs to inhibit these changes in the lungs were assessed using HE (Fig. 3a) and Masson’s trichrome staining (Fig. 3b). The mean linear intercept (Fig. 3c) and mean alveolar volume (Fig. 3d) were calculated to quantitatively analyse the level of alveolar expansion according to HE staining. CLP morphologically and quantitatively induced alveolar expansion, and MSCs relieved these pathological changes (Fig. 3a, c and d). Several other pathological changes induced by CLP were observed morphologically, including irregular changes in the bronchial tube and interstitial hyperplasia, and MSCs partially reversed these histological changes (Fig. 3b). CLP-induced lung oedema, as assessed by the wet to dry weight ratio, was reduced by both MSC treatments (Fig. 3e). TUNEL staining was performed to label dead cells in the lung (Fig. 3f). MSCs from both sources significantly reduced the number of TUNEL-positive cells compared with the sepsis group, and no significant differences were observed between the AF-MSC- and UC-MSC-treated groups (Fig. 3g). The levels of the proinflammatory cytokine IL-1β, IL-10 and TNF-α in the BALF were assessed (Fig. 3h–j). MSC treatments reduced the CLP-induced increase in the levels of these cytokines, and significantly lower levels of these three cytokines were detected in the UC-MSC group than in the AF-MSC group.

### MSCs attenuate CLP-induced pathological change of capsule and glycogen consumption in liver

**Fig. 4:**
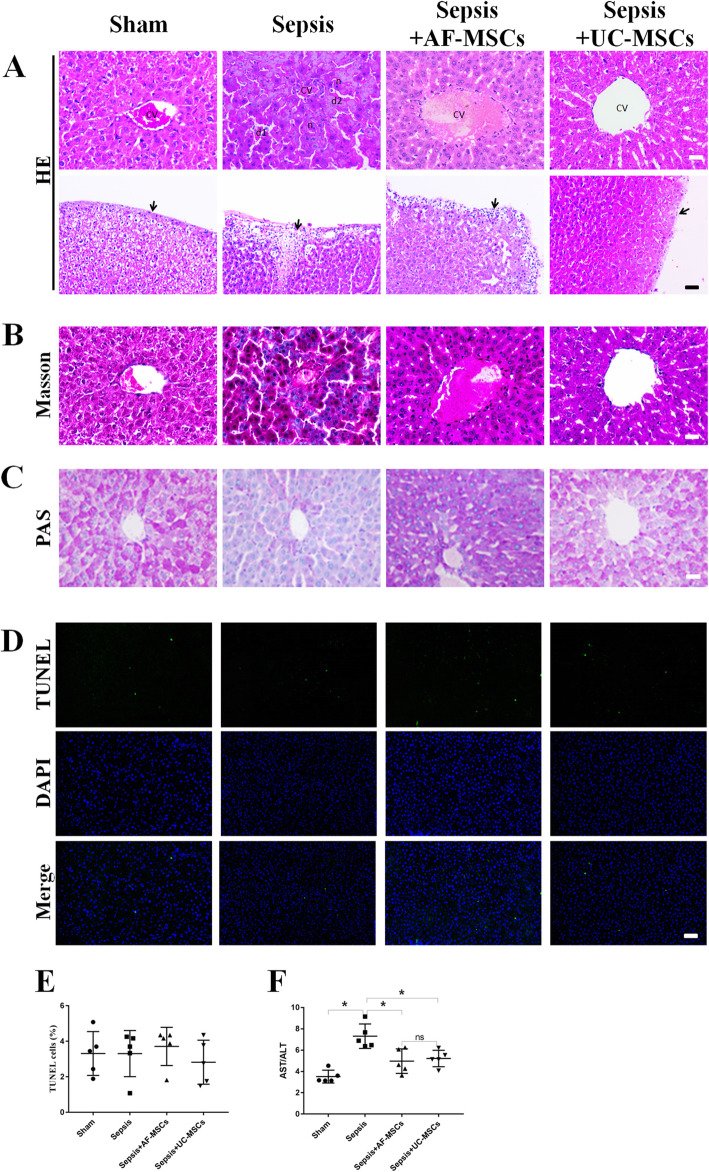
The administration of MSCs improved CLP-induced histological and functional changes in the liver of septic rats, while UC-MSCs performed better in reducing inflammatory cell infiltration than AF-MSCs. The histological changes in the liver were identified by performing HE (**a**), Masson’s trichrome (**b**) and PAS (**c**) staining. In **a**, a representative image of the liver CV is shown in the upper panel, and the capsule is shown in the lower panel. Necrotic and degenerative hepatocytes were observed near the CV, and inflammatory cell infiltration into the capsule (black arrow) was evident in the sepsis group. The administration of MSCs improved CLP-induced cell death (as determined by the morphology), capsule inflammation, swelling of vascular endothelial cells and reduction in glycogen levels within hepatocytes, while inflammatory cell infiltration was still present in the AF-MSC group. No difference in cell death in the liver was detected among the four groups, as assessed using TUNEL staining (**d** and **e**). Other assessments of liver function included measurements of the AST/ALT ratio (**f**). Both types of MSCs relieved the CLP-induced increase in this parameter. CV: central vein; n: necrotic hepatocytes with an eosinophilic cytoplasm and pyknotic nucleus; d1, degenerating cells with a loss of the intact cell morphology; d2, degenerative area with understained cytoplasm. For images shown in the upper panels of **a**, **b** and **c**, scale bar = 20 μm; for images shown in the lower panels of **a**, scale bar = 50 μm; for **d**, scale bar = 100 μm. *p < 0.05; ns, no significant difference

CLP-induced sepsis causes histological and biochemical changes in the liver, which were equally attenuated by AF-MSC and UC-MSC administration. In the sepsis group, a few necrotic hepatocytes with an eosinophilic cytoplasm and a pyknotic nucleus, degenerating cells with considerably intact cell morphology and a degenerative area of cytoplasm weakly stained with HE were observed, while the histology of both of the MSC groups was more homogeneous and similar to the sham group, without any understained areas (Fig. 4a). Regarding inflammation, sepsis caused inflammatory cell infiltration that affected the capsule and parenchyma of the liver. Some inflammatory cell infiltration of the liver capsule was evident in the AF-MSC group but was rarely observed in the UC-MSC group, according to HE staining (Fig. 4a). In terms of other histological changes, the MSC groups showed improvements in the CLP-induced swelling of the vascular endothelial cells, as detected using HE and Masson’s trichrome staining, compared with the sepsis group (Fig. 4a and b). Both MSC treatments relieved the CLP-induced reduction in liver glycogen levels (Fig. 4c). A significant difference in the number of TUNEL-labelled dead cells in the liver was not observed among the four groups (Fig. 4d and e). Sepsis induced a significant increase in the AST to ALT ratio, which was significantly decreased by both MSC treatments, but a significant difference was not observed between the AF-MSC and UC-MSC groups (Fig. 4f).

### MSCs improve sepsis-associated pathological changes and cell death in the kidney

**Fig. 5:**
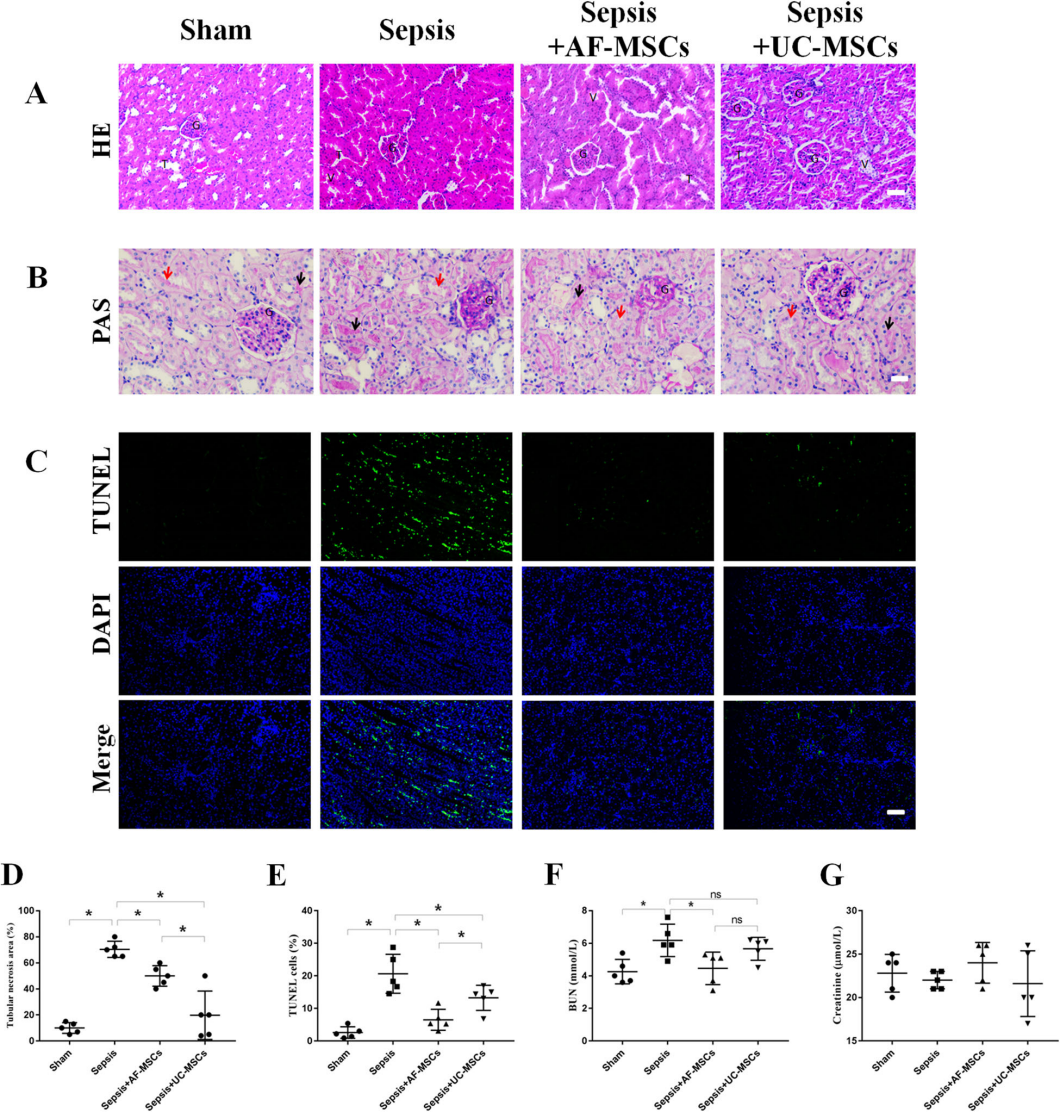
The administration of MSCs improved CLP-induced pathological changes in the kidneys of septic rats, while AF-MSCs more effectively reduced cell death and BUN levels than UC-MSCs. The histological changes in the kidney were identified by performing HE (**a**) and PAS (**b**) staining, while cell death was determined by performing TUNEL staining (**c**). The administration of MSCs improved CLP-induced histological changes (including vacuolization and oedema of the glomeruli and tubules) and cell death, while UC-MSCs more substantially reduced tubular necrosis, as evidenced by PAS staining (**d**, red arrow for normal tubules and black arrow for tubular lumens with a loss of brush borders), and AF-MSCs reduced cell death to a greater extent, as evidenced by TUNEL staining (**e**). Other assessment of biochemical parameters related to kidney function included measurements of BUN (**f**) and creatinine (**g**) levels. CLP increased BUN levels, a change that was improved by AF-MSCs but not by UC-MSCs; no significant difference in creatinine levels was observed among the four groups. **a**, scale bar = 200 μm; **b**, scale bar = 100 μm; **c**, scale bar = 100 μm. G, glomerulus; T, tubule; V, vacuolization. *p < 0.05; ns, no significant difference

CLP-induced sepsis causes histological changes, cell death and biochemical changes within the kidney. The administration of MSCs attenuated the sepsis-associated phenotype, and AF-MSCs were more effective than UC-MSCs. Histology of the healthy kidney revealed normal glomerular and tubular structures in the sham group. In the sepsis model, pathological changes in the glomeruli were not typically observed, but some histological changes in tubular structures were evident, including epithelial swelling, loss of the brush border of luminal cells and the appearance of vacuolated areas. Prominent improvements induced by MSC therapy included a reversal of the tubular swelling and the observation of a normal morphology of the endothelial cells (Fig. 5a). Both MSC therapies reduced the CLP-induced increase in the tubular necrosis area (Fig. 5b) and TUNEL-positive cells (Fig. 5c) in the kidney, and the AF-MSCs resulted in a significantly larger tubular necrosis area (Fig. 5d) and smaller TUNEL-positive cell ratio (Fig. 5e) than the UC-MSCs. Serum BUN and creatinine levels were measured as indicators of CLP-induced kidney function damage. Compared with the sepsis group, the AF-MSC group, but not the UC-MSC group, exhibited significantly decreased BUN levels (Fig. 5d), but a significant difference in creatinine levels was not observed among the four groups (Fig. 5e).

## Discussion

The CLP rodent model is the most commonly used animal model in sepsis studies [18]. After CLP surgery, the intestinal pathogens, which initiate polymicrobial and mainly bacterial infections, escape to the abdominal cavity and trigger a systemic inflammatory response via the blood circulation. The severity of CLP-induced sepsis is predominantly increased at the puncture sites [17]. In our study, we generated rats with medium-grade sepsis and a 30–40% survival rate to study the curative effects of AF-MSCs and UC-MSCs. Based on previously reported research experience [19], we chose 6 h after CLP to perform our analyses. As few GFP-labelled MSCs were detected in the lung and brain, where the therapeutic effects of MSC were observed, this indicated that prophylactic treatment with MSC could systematically reduce the inflammatory damages in the whole body [20–22]. Furthermore, because the circulatory system and cellular microenvironment are the main battlefield of the inflammatory response [19], after intravenous administration of cells, most of these cells are dead and consumed, mainly due to an acute immune response, and few cells are located in the effector organs to exert their effects. Therefore, our research mainly detects the inflammatory response of cell therapy by biochemical assay to determine the different curative effects of AF-MSCs or UC-MSCs in different tissues, but not tracking the location of the AF-MSCs or UC-MSCs in vivo by fluorescent flag.

Safe administration of MSCs can be determined by using certain therapeutic schedules. For the clinical usage of cytotherapy, the first requirement is safety, and some concerns have been noted regarding the administration of MSCs. Reports of brachial vein [23] and pulmonary [24] thromboembolism induced by MSC infusion have been documented. Animal studies have also shown that an overdose of MSCs is lethal [25] if MSCs accumulate in the pulmonary circulation [26, 27], leading to thromboembolism [25]. Notably, these reports examined MSCs derived from adipose tissue and umbilical cord. MSCs from these two sources are the most widely used MSCs; however, some differences may exist among MSCs derived from different tissues, including cell size, cell surface markers and cellular biofunctions. In our previous study, different culture method changing MSC molecule marker but not affecting differentiation ability or immune function, and AF-MSCs showed similar bio-function with UC-MSCs in vitro [16], so we tested the bio-function of AF-MSCs and UC-MSCs in CLP-induced rat sepsis in this study and found out that removal of agglomerated UC-MSCs was very important for its safety usage. Previous study had confirmed that tissue factor [6] has a critical role in promoting MSC-mediated coagulation in living animals and that its expression is likely to lead to thromboembolism and subsequent death of a patient treated with MSCs [25], as TF is highly expressed at the level of mRNA and localized to the cell surface of cultured MSCs, a triggering factor in the procoagulative cascade activated by infused MSCs [25]. In our study, the survival rate of unfiltered AF-MSCs was significantly higher than that of unfiltered UC-MSCs, indicating that unfiltered UC-MSCs may had high dose of TF. Furthermore, the survival rate was not significantly different between filtered AF-MSCs’ and filtered UC-MSCs’ treatments in our study which also indicated that filtered AF-MSCs and UC-MSCs produced a similar curative effect in terms of CLP-induced sepsis, but exerted different bioeffects on different tissues.

Based on criteria from the ISCT [28], MSCs can be derived from nearly all types of postpartum and antenatal organs, including adipose tissue, menstrual fluid, umbilical cord (Wharton’s jelly), fat, placenta and muscle, and MSCs from different sources possess similar but not identical functions. The convenience of these sources has promoted the usage of MSCs for cytotherapy. The umbilical cord is discarded after most obstetric procedures, and therefore, cytotherapy with UC-MSCs can be regarded as waste utilization. Amniotic fluid is generated by the foetal circulatory system during foetal growth and development. Sepsis is a life-threatening disorder of organ dysfunction that is characterized by sustained excessive inflammation and immune suppression, and the therapeutic effect of MSCs has been attributed to its anti-inflammatory properties in vivo and in vitro [1]. Although the biofunction mechanism of MSCs from human and rodent was different, even human MSCs were not activated by rat pro-inflammatory cytokines in corneal allograft model [29], human MSCs’ therapeutic efficacy to rat CLP induced sepsis were confirmed by other study [19], the most reasonable possibility was due to inflammation affect tissue was different, so the MSCs’ biofunction in sepecific tissue has a high study value. In the present study, both UC-MSCs and AF-MSCs improved the survival rate of septic rats from 30 to 60%, reduced tissue inflammation and attenuated damage to the lung, liver and kidney, as determined by the biochemical and histological analyses of the rats with CLP-induced sepsis, consistent with the confirmed therapeutic effects of other MSCs [10].

The curative effects of AF-MSCs and UC-MSCs on rats with CLP-induced sepsis differed. UC-MSCs are one of the most widely used cell types in cytotherapy, and their bioeffect on curing sepsis has been described in several studies [19, 30–32], but few reports have assessed sepsis cytotherapy utilizing AF-MSCs, except in a neonatal rat model of sepsis [33]. The ability of the AF-MSC treatment to reduce local inflammation in rodent models of perinatal diseases, such as hypoxic ischaemic encephalopathy and foetal myelomeningocele, has been reported [34, 35]. Thus, foetal stem cells produced during pregnancy may be used for neonatal autologous somatic therapy if needed immediately after birth or during pregnancy [36]. According to previous studies, bone marrow-derived mesenchymal stem cells (BMSCs) are more effective than adipose-derived mesenchymal stem cells (ADMSCs) as a treatment for experimental sepsis in mice [37]. In vitro studies showed that umbilical cord blood-derived mesenchymal stem cells were superior to BMSCs and ADMSCs in reducing the production of proinflammatory cytokines [38]. Additional in vitro studies have shown that MSCs derived from the umbilical cord matrix, known as Wharton’s jelly-derived MSCs (WJ-MSCs), present characteristics similar to or better than other MSCs and show promise as a treatment for various diseases [39, 40]. AF-MSCs are derived from multiple tissue sources and express higher levels of lung- and kidney-specific markers, with enhanced osteogenic and chrondrogenic differentiation and a similar immunosuppression ability to UC-MSCs [41]. In the present study, we described the different bioeffects of AF-MSCs and UC-MSCs on different tissues from rats with CLP-induced sepsis. UC-MSCs performed better in immunosuppression following CLP induction than AF-MSCs, including reducing the upregulation of proinflammatory cytokine levels in the lung and inhibition of the inflammatory cell infiltration into the liver capsule. In sepsis, an initial pro-inflammatory phase is characterized by increased cytokine levels and a CD4+ Th1 cell response. In the present study, UC-MSC and AF-MSC treatments improved tissue inflammation and attenuated LPS-induced damage to the lung, liver and kidney, as determined by the histological analysis, consistent with the reported therapeutic effects of other MSCs [10, 19, 42, 43]. In our study, the animals with sepsis developed proinflammatory profiles that were characterized by increased expression of IL-1β, IL-10, IL-6 and TNF-α. Treatment with UC-MSCs and AF-MSCs decreased the expression of those cytokines. AF-MSCs were the most effective treatment at rescuing kidney biofunction, including the inhibition of cell death and reduction of plasma BUN levels (an indicator of renal function), potentially due to the ability of AF-MSCs to accelerate the proliferation of partially damaged epithelial tubular cells and prevent apoptosis [44].

The beneficial effect of AF-MSCs was probably related to the paracrine action of secreted growth factors [45]. A WJ-MSC treatment was reported to induce a pronounced increase in VEGF expression and reversed the sepsis-induced downregulation of eNOS expression [19]. Sepsis is a model of endothelial dysfunction, and eNOS and VEGF protect against sepsis-induced endothelial dysfunction [46, 47] and are effective at normalizing liver enzymes, mainly AST [48]. MSCs have been suggested to protect against sepsis-induced liver injury by decreasing leukocyte infiltration [49, 50], modulating the immune response and decreasing apoptosis [51]. Thus, the molecular mechanisms underlying the different curative effects of AF-MSCs and UC-MSCs are likely different and require further study.

## Conclusion

In summary, in addition to highlighting that different cell production processes may lead to diverse physicochemical properties, biological characters and therapeutic effects of MSCs, our studies have identified the safety and efficacy of AF-MSCs and UC-MSCs in the healing of rats with CLP-induced sepsis and their potential differences in ameliorating the effects of sepsis on different tissues. Further preclinical studies are needed to define the molecular variations in MSCs derived from different sources via different production schemes, to establish the relationship between the cell phenotype in vitro and the cytotherapeutic biofunctions of MSCs in vivo, to improve the curative effects of MSCs and to obtain a better understanding of the differences in the curative effects of MSCs from different tissue sources.

## Data Availability

All data generated or analysed during this study are included in this published article (and its supplementary information files).

## Abbreviations

SIRS: Systemic inflammatory response syndrome
MSCs: Mesenchymal stem cells
AF-MSCs: Amniotic fluid-MSCs
UC-MSCs: Umbilical cord-MSCs
CLP: Caecal ligation and puncture
HE: Haematoxylin–eosin
TUNEL: TdT-mediated dUTP nick-end labelling
BALF: Bronchoalveolar lavage fluid
ELISA: Enzyme-linked immunosorbent assay
PT: Prothrombin time
APTT: Activated partial thromboplastin time
FIB: Fibrinogen
AST: Aspartate aminotransferase
ALT: Alanine aminotransferase
BUN: Blood urea nitrogen

## References

[1] van der Poll T, van de Veerdonk FL, Scicluna BP, Netea MG (2017). The immunopathology of sepsis and potential therapeutic targets. Nat Rev Immunol.

[2] Abraham E, Singer M (2007). Mechanisms of sepsis-induced organ dysfunction. Crit Care Med.

[3] Rhodes A, Evans LE, Alhazzani W, Levy MM, Antonelli M, Ferrer R, Kumar A, Sevransky JE, Sprung CL, Nunnally ME (2017). Surviving Sepsis Campaign: International Guidelines for Management of Sepsis and Septic Shock: 2016. Crit Care Med.

[4] Gaieski DF, Edwards JM, Kallan MJ, Carr BG (2013). Benchmarking the incidence and mortality of severe sepsis in the United States. Crit Care Med.

[5] Kaukonen KM, Bailey M, Suzuki S, Pilcher D, Bellomo R (2014). Mortality related to severe sepsis and septic shock among critically III patients in Australia and New Zealand, 2000-2012. JAMA.

[6] Arad G, Levy R, Nasie I, Hillman D, Rotfogel Z, Barash U, Supper E, Shpilka T, Minis A, Kaempfer R. Binding of superantigen toxins into the CD28 homodimer interface is essential for induction of cytokine genes that mediate lethal shock. PLoS Biol. 2011;9(9):e1001149. 10.1371/journal.pbio.1001149 PMC3172200 21931534

[7] Unsinger J, McGlynn M, Kasten KR, Hoekzema AS, Watanabe E, Muenzer JT, McDonough JS, Tschoep J, Ferguson TA, McDunn JE (2010). IL-7 promotes T cell viability, trafficking, and functionality and improves survival in Sepsis. J Immunol.

[8] Docke WD, Randow F, Syrbe U, Krausch D, Asadullah K, Reinke P, Volk HD, Kox W (1997). Monocyte deactivation in septic patients: restoration by IFN-gamma treatment. Nat Med.

[9] Shao R, Fang YY, Yu H, Zhao LX, Jiang ZF, Li CS. Monocyte programmed death ligand-1 expression after 3-4 days of sepsis is associated with risk stratification and mortality in septic patients: a prospective cohort study. Crit Care. 2016;20(1):124. 10.1186/s13054-016-1301-x PMC4860759 27156867

[10] Laroye C, Gibot S, Reppel L, Bensoussan D (2017). Concise review: Mesenchymal stromal/stem cells: a new treatment for sepsis and septic shock?. Stem Cells.

[11] Caplan AI, Correa D (2011). The MSC: an injury drugstore. Cell Stem Cell.

[12] Bianco P, Robey PG, Saggio I, Riminucci M (2010). “Mesenchymal” stem cells in human bone marrow (skeletal stem cells): a critical discussion of their nature, identity, and significance in incurable skeletal disease. Hum Gene Ther.

[13] Li N, Hua JL (2017). Interactions between mesenchymal stem cells and the immune system. Cell Mol Life Sci.

[14] Naji A, Favier B, Deschaseaux F, Rouas-Freiss N, Eitoku M, Suganuma N (2019). Mesenchymal stem/stromal cell function in modulating cell death. Stem Cell Res Ther.

[15] Coelho A, Alvites RD, Branquinho MV, Guerreiro SG, Mauricio AC (2020). Mesenchymal stem cells (MSCs) as a potential therapeutic strategy in COVID-19 patients: literature research. Front Cell Dev Biol.

[16] Wang D, Liu N, Xie Y, Song B, Kong S, Sun X (2020). Different culture method changing CD105 expression in amniotic fluid MSCs without affecting differentiation ability or immune function. J Cell Mol Med.

[17] Rittirsch D, Huber-Lang MS, Flierl MA, Ward PA (2009). Immunodesign of experimental sepsis by cecal ligation and puncture. Nat Protoc.

[18] Buras JA, Holzmann B, Sitkovsky M (2005). Animal models of sepsis: setting the stage. Nat Rev Drug Discov.

[19] Condor JM, Rodrigues CE, Sousa Moreira R, Canale D, Volpini RA, Shimizu MH, Camara NO, Noronha Ide L, Andrade L (2016). Treatment with human Wharton’s jelly-derived mesenchymal stem cells attenuates sepsis-induced kidney injury, liver injury, and endothelial dysfunction. Stem Cells Transl Med.

[20] Sala E, Genua M, Petti L, Anselmo A, Arena V, Cibella J, Zanotti L, D'Alessio S, Scaldaferri F, Luca G (2015). Mesenchymal stem cells reduce colitis in mice via release of TSG6, independently of their localization to the intestine. Gastroenterology.

[21] Bazhanov N, Ylostalo JH, Bartosh TJ, Tiblow A, Mohammadipoor A, Foskett A, Prockop DJ (2016). Intraperitoneally infused human mesenchymal stem cells form aggregates with mouse immune cells and attach to peritoneal organs. Stem Cell Res Ther.

[22] Song WJ, Li Q, Ryu MO, Ahn JO, Bhang DH, Jung YC, Youn HY (2018). TSG-6 released from intraperitoneally injected canine adipose tissue-derived mesenchymal stem cells ameliorate inflammatory bowel disease by inducing M2 macrophage switch in mice. Stem Cell Res Ther.

[23] Wu Z, Zhang S, Zhou L, Cai J, Tan J, Gao X, Zeng Z, Li D (2017). Thromboembolism induced by umbilical cord mesenchymal stem cell infusion: a report of two cases and literature review. Transplant Proc.

[24] Mizuno H (2010). Adipose-derived stem and stromal cells for cell-based therapy: current status of preclinical studies and clinical trials. Curr Opin Mol Ther.

[25] Tatsumi K, Ohashi K, Matsubara Y, Kohori A, Ohno T, Kakidachi H, Horii A, Kanegae K, Utoh R, Iwata T (2013). Tissue factor triggers procoagulation in transplanted mesenchymal stem cells leading to thromboembolism. Biochem Biophys Res Commun.

[26] Furlani D, Ugurlucan M, Ong L, Bieback K, Pittermann E, Westien I, Wang W, Yerebakan C, Li W, Gaebel R (2009). Is the intravascular administration of mesenchymal stem cells safe? Mesenchymal stem cells and intravital microscopy. Microvasc Res.

[27] Gao J, Dennis JE, Muzic RF, Lundberg M, Caplan AI (2001). The dynamic in vivo distribution of bone marrow-derived mesenchymal stem cells after infusion. Cells Tissues Organs.

[28] Dominici M, Le Blanc K, Mueller I, Slaper-Cortenbach I, Marini F, Krause D, Deans R, Keating A, Prockop D, Horwitz E (2006). Minimal criteria for defining multipotent mesenchymal stromal cells. The International Society for Cellular Therapy position statement. Cytotherapy.

[29] Lohan P, Treacy O, Morcos M, Donohoe E, O'Donoghue Y, Ryan AE, Elliman SJ, Ritter T, Griffin MD (2018). Interspecies incompatibilities limit the immunomodulatory effect of human mesenchymal stromal cells in the rat. Stem Cells.

[30] Chao YH, Wu HP, Wu KH, Tsai YG, Peng CT, Lin KC, Chao WR, Lee MS, Fu YC (2014). An increase in CD3+CD4+CD25+ regulatory T cells after administration of umbilical cord-derived mesenchymal stem cells during sepsis. PLoS One.

[31] Zhao X, Liu D, Gong W, Zhao G, Liu L, Yang L, Hou Y (2014). The toll-like receptor 3 ligand, poly(I:C), improves immunosuppressive function and therapeutic effect of mesenchymal stem cells on sepsis via inhibiting MiR-143. Stem Cells.

[32] Song Y, Dou H, Li X, Zhao X, Li Y, Liu D, Ji J, Liu F, Ding L, Ni Y (2017). Exosomal miR-146a contributes to the enhanced therapeutic efficacy of interleukin-1beta-primed mesenchymal stem cells against sepsis. Stem Cells.

[33] Sato Y, Ochiai D, Abe Y, Masuda H, Fukutake M, Ikenoue S, Kasuga Y, Shimoda M, Kanai Y, Tanaka M (2020). Prophylactic therapy with human amniotic fluid stem cells improved survival in a rat model of lipopolysaccharide-induced neonatal sepsis through immunomodulation via aggregates with peritoneal macrophages. Stem Cell Res Ther.

[34] Otani T, Ochiai D, Masuda H, Abe Y, Fukutake M, Matsumoto T, Miyakoshi K, Tanaka M (2019). The neurorestorative effect of human amniotic fluid stem cells on the chronic phase of neonatal hypoxic-ischemic encephalopathy in mice. Pediatr Res.

[35] Abe Y, Ochiai D, Masuda H, Sato Y, Otani T, Fukutake M, Ikenoue S, Miyakoshi K, Okano H, Tanaka M (2019). In utero amniotic fluid stem cell therapy protects against myelomeningocele via spinal cord coverage and hepatocyte growth factor secretion. Stem Cells Transl Med.

[36] Ochiai D, Masuda H, Abe Y, Otani T, Fukutake M, Matsumoto T, Miyakoshi K, Tanaka M (2018). Human amniotic fluid stem cells: therapeutic potential for perinatal patients with intractable neurological disease. Keio J Med.

[37] Elman JS, Li M, Wang F, Gimble JM, Parekkadan B (2014). A comparison of adipose and bone marrow-derived mesenchymal stromal cell secreted factors in the treatment of systemic inflammation. J Inflamm.

[38] Jin HJ, Bae YK, Kim M, Kwon SJ, Jeon HB, Choi SJ, Kim SW, Yang YS, Oh W, Chang JW (2013). Comparative analysis of human mesenchymal stem cells from bone marrow, adipose tissue, and umbilical cord blood as sources of cell therapy. Int J Mol Sci.

[39] Romanov YA, Svintsitskaya VA, Smirnov VN (2003). Searching for alternative sources of postnatal human mesenchymal stem cells: candidate MSC-like cells from umbilical cord. Stem Cells.

[40] Watson N, Divers R, Kedar R, Mehindru A, Mehindru A, Borlongan MC, Borlongan CV (2015). Discarded Wharton jelly of the human umbilical cord: a viable source for mesenchymal stromal cells. Cytotherapy.

[41] Loukogeorgakis SP, De Coppi P (2017). Concise review: Amniotic fluid stem cells: the known, the unknown, and potential regenerative medicine applications. Stem Cells.

[42] Yagi H, Soto-Gutierrez A, Kitagawa Y, Tilles AW, Tompkins RG, Yarmush ML (2010). Bone marrow mesenchymal stromal cells attenuate organ injury induced by LPS and burn. Cell Transplant.

[43] Zhu Y, Xu L, Collins JJP, Vadivel A, Cyr-Depauw C, Zhong S, Mense L, Mobius MA, Thebaud B (2017). Human umbilical cord mesenchymal stromal cells improve survival and bacterial clearance in neonatal sepsis in rats. Stem Cells Dev.

[44] Perin L, Sedrakyan S, Giuliani S, Da Sacco S, Carraro G, Shiri L, Lemley KV, Rosol M, Wu S, Atala A (2010). Protective effect of human amniotic fluid stem cells in an immunodeficient mouse model of acute tubular necrosis. PLoS One.

[45] Pederiva F, Ghionzoli M, Pierro A, De Coppi P, Tovar JA (2013). Amniotic fluid stem cells rescue both in vitro and in vivo growth, innervation, and motility in nitrofen-exposed hypoplastic rat lungs through paracrine effects. Cell Transplant.

[46] Jesmin S, Zaedi S, Islam AM, Sultana SN, Iwashima Y, Wada T, Yamaguchi N, Hiroe M, Gando S (2012). Time-dependent alterations of VEGF and its signaling molecules in acute lung injury in a rat model of sepsis. Inflammation.

[47] Xu C, Chang A, Hack BK, Eadon MT, Alper SL, Cunningham PN (2014). TNF-mediated damage to glomerular endothelium is an important determinant of acute kidney injury in sepsis. Kidney Int.

[48] Chang CL, Leu S, Sung HC, Zhen YY, Cho CL, Chen A, Tsai TH, Chung SY, Chai HT, Sun CK (2012). Impact of apoptotic adipose-derived mesenchymal stem cells on attenuating organ damage and reducing mortality in rat sepsis syndrome induced by cecal puncture and ligation. J Transl Med.

[49] Rochefort GY, Delorme B, Lopez A, Herault O, Bonnet P, Charbord P, Eder V, Domenech J (2006). Multipotential mesenchymal stem cells are mobilized into peripheral blood by hypoxia. Stem Cells.

[50] Gonzalez-Rey E, Anderson P, Gonzalez MA, Rico L, Buscher D, Delgado M (2009). Human adult stem cells derived from adipose tissue protect against experimental colitis and sepsis. Gut.

[51] Mei SH, Haitsma JJ, Dos Santos CC, Deng Y, Lai PF, Slutsky AS, Liles WC, Stewart DJ (2010). Mesenchymal stem cells reduce inflammation while enhancing bacterial clearance and improving survival in sepsis. Am J Respir Crit Care Med.

